# Gender Affirmative Action and Management: A Systematic Literature Review on How Diversity and Inclusion Management Affect Gender Equity in Organizations

**DOI:** 10.3390/bs11020021

**Published:** 2021-02-04

**Authors:** Julia V. Furtado, António C. Moreira, Jorge Mota

**Affiliations:** 1 Departamento de Gestão e Economia, Universidade da Beira Interior, 6201-001 Covilhã, Portugal; 2 Department of Economics, Management and Industrial Engineering and Tourism, GOVCOPP, Universidade de Aveiro, 3810-193 Aveiro, Portugal; amoreira@ua.pt (A.C.M.); jorgemota@ua.pt (J.M.)

**Keywords:** gender affirmative action, job satisfaction, social dominance orientation, organizational commitment, diversity management

## Abstract

Gender affirmative action (AA) in management remains a controversial topic among scholars, practitioners, and employees. While some individuals may support the use of AA policies as a means of increasing representation of women, others are not supportive at all, further understanding gender AA as an unacceptable violation of merit—even when targeted by it. With the aim of analyzing how scholars have approached the subject, we systematically reviewed 76 published articles (SCOPUS database), covering the extant literature on gender AA and management. Findings indicate a consensus regarding the common antecedents of attitudes towards gender AA with prior experiences with AA and diversity management (DM) (as well as general perceptions of AA). Performance and satisfaction appear as the predominant outcomes. In addition, while investigating the differences among AA, equal employment opportunity (EEO) and diversity management (DM), scholars are mainly focused on the effectiveness of AA as a means of increasing the inclusion of minorities in general. We conclude that despite marginal studies on employees’ attitudes toward gender AA, there is a gap in the literature, particularly an absence of research on the bivalent position of meritocracy (or merit violation) as both an antecedent and outcome of attitudes towards AA, which deserves further scrutiny.

## 1. Introduction

Gender equality and empowerment of all women and girls is one of the 17 goals of the UN 2030 Agenda for Sustainable Development, which comprises 169 targets in a universal plan of action for people, planet, and prosperity [1]. The UN Secretary-General António Guterres has consistently focused on the long-standing demand by women for gender equality, in what he argues to be “the unfinished business of our time” [2]. Henceforth, the goal of gender parity, defined as the equal participation of women and men in positions of power and decision-making [3], has gained increased attention globally over the past decades. 

Starting in 1975, with the United Nations General Assembly (UN) proclaiming 1976–1985 as the UN Decade for Women, several UN resolutions targeted an increase in the proportion of women in leadership positions [4,5]. The spread of a myriad of affirmative action (AA) and equal employment opportunity (EEO) initiatives was also promoted with the aim of reducing the underrepresentation of women (and minorities in general) both in politics and leadership positions. However, the effects of such actions are equally diversified. 

Fostering equality among men and women has been part of the European Union’s founding values, promoting the principle of “equal pay for equal work” between genders, which has been part of the European Treaties since 1957 (currently Article 157 of the Treaty on the Functioning of the European Union (TFEU) [6]. Actions, however, have developed beyond employment opportunities in entry positions. Indeed, the EU has consistently nurtured equality between genders through the promotion of equal opportunities for men and women either in corporate board representation and decision-making or on other workplace levels [7]. 

Moreover, the debate over gender diversity on boards has captured attention around the world. Norway, for instance, directed a gender quota for boards of directors (40% of women), followed by Spain and France [8]. Sweden, on the other hand, has assigned quotas of 25% of female representation on corporate boards. However, the enforcement of gender quota for boards is not a global initiative. In stark contrast to the aforementioned European nations, Australia has no mandatory gender quotas for boards [8], with soft quotas or voluntary actions being more common initiatives. 

Australian studies on gender equity initiatives have shown that initiatives reinforcing the existing gender order in society (responding to women’s immediate needs as wives and mothers) are quickly accepted, while actions that challenge the existing gender order face low acceptance [9]. In terms of gender attitudinal differences, women tend to be more supportive of gender parity initiatives and equalitarian intergroup relationships than men [9,10]. Reservations, doubts, and qualifications on gender equity programs are common in both genders [3,9,10,11]. It is precisely this diversity of understandings, perceptions, and attitudes concerning gender AA within organizations that ignited the motivation for this study. In an attempt to shed light on perceptions of gender AA, especially among employees, a thorough analysis of how scholars have approached the subject over the years had been employed.

Although not a particularly new topic, gender equality initiatives are central to both the UN 2030 Agenda for Sustainable Development and European Union Treaties. Concern about gender parity, as well as minority inclusion, has gained increased attention, particularly following the announcement of UN goals and OECD recommendations. Scholars have paid particular attention to diversity inclusion and management via AA and its effects on organizations. The number of studies, however, is still limited, and indicates a potential combination of different antecedents influencing attitudes and behavior towards AA, particularly gender equity initiatives. Hence, the need for further studies compiling the most relevant antecedents and outcomes of gender AA in organizations becomes evident. Filling this gap in the management literature not only expands the discussion about the need for gender AA and EEO, but also provides further scrutiny of how diversity and inclusion management affects gender equity in organizations. 

The main objective of this systematic literature review (SLR) was to map the state of the art in extant management literature, analyzing gender affirmative action and management, particularly concerning employees’ attitudes towards affirmative action for gender parity in organizations. Therefore, a contemporary theoretical background of affirmative action, equal employment opportunity, and diversity management is necessary. 

### 1.1. Affirmative Action

Apparently similar, affirmative action (AA), equal employment opportunities (EEO) and diversity management (DM) are different and somewhat interdependent concepts [12,13]. Affirmative action (AA) is understood as initiatives which simply compensate for societal barriers that hinder women from having equal access to representation [14,15]. Or from a different perspective, AA attempts to redress past disadvantages and disparate labor outcomes for minorities—women included [12].

Gender quotas or gender AA can be defined as initiatives meant to improve women’s presence and representation in legislature, government, and industry. The most common mechanisms for increasing women’s participation both in politics and decision-making positions are electoral and corporative quotas, implying a mandatory percentage of women in leadership positions. It typically involves creating a percentage target for the representation of women, as a group who have been historically excluded or underrepresented [15]. While varying among countries and organizations, gender quotas—via AA or DM—usually aim to produce a “critical mass” of female candidates and leaders [16].

In politics, scholars commonly identify four basic types of quotas as enforcement mechanisms to assure access to political office for women: party quotas, legislative quotas, reserved seats, and soft quotas [16], the first three being known as “hard quotas” because they require parties and governments to nominate between 25 and 50 percent of women candidates and reserved seats in a government [17]. Soft quotas include informal targets or policies that encourage parties or organizations to include more women in their nomination processes, without any formal obligation [15]. The idea of quotas for women either in politics or in leadership positions in industry remains controversial, with men and women sustaining the argument that “women must ‘make it’ according to the time-honored rules of the game because this test alone determines genuine merit” [18] (p. 88). 

Nevertheless, rather than promoting substantive change, quotas and AA for gender parity may create an atmosphere where women are not taken seriously [14,15]. Studies on AA at the organizational level have connected paradoxical results of such plans, concerning initiatives designed to enable workplace success for underrepresented groups. The results have shown that these organizational actions may backfire, not only stigmatizing members of the groups they target (e.g., women) but also reducing their outcomes in terms of performance [19]. 

Based on a qualitative study with 60 male and female directors (in the U.S. and Europe), Wiersema and Mors [11] investigated the perceptions of gender-based quotas on corporate boards and revealed hostility toward quotas, particularly in countries that do not have them at the political level (Denmark and U.S.). One of the reasons identified was the belief that “it would lead to the selection of unqualified women or selection purely on gender” [11] (p. 3). In those countries where AA at the organizational level is combined with the government imposing quotas and goals of gender equity, such as in Norway, not only has greater gender diversity been reached, but it has also led to more professional and formal approaches to board selection, resulting in higher support among the CEOs interviewed [11].

Further research on support for quotas has shown that the effects of gender AA plans depend on whether these policies are viewed favorably within those potentially affected groups [20]. For instance, a gender quota would be acceptable for individuals at the organizational level if justified by a reasonable historical rationale—such as EEO policies in South Africa as amendments to apartheid events in the past that prevented black women’s access to job opportunities, for instance—but only in the presence of such historical justification [20,21].

Additionally, while some men are strong supporters of gender equality, men as a group have a lower level of knowledge about gender equity programs and are less likely to support them [9]. Quite common also is the belief that “there is a current policy of favoring the appointment of women above better-qualified men” present in the responses from people of both genders [9] (p. 149). 

Lastly, some authors agree that AA initiatives in management, such as the use of gender quotas, can be very controversial, with employees presenting opposite attitudes towards them [9,10,11,22,23]. Some may actively support the use of AA as a means of increasing women’s descriptive representation, while others are not supportive at all. Niederle et al. [24] argue that whenever AA is put in place seeking gender parity, entry by women increases while entry by men inversely decreases. The implicit idea here would be that instead of reaching equity by giving access to women, it might be achieved by reducing men’s presence—clear evidence of reverse discrimination. Beyond that, whenever employees are not convinced of the need for gender AA, significant levels of cynicism and mistrust arise, influencing their individual attitudes [25].

Finally, authors agree in contextualizing AA as problem-oriented and accountable— or law legislated—initiatives [12,26,27,28,29]. Allen et al. [26] imply that AA targets per se do not effectively improve the perception of diversity—or attitudes towards it—within the managerial levels of organizations. Instead, critical initiatives such as internships, mentoring and support networks for minorities are expected to positively influence the perception of managerial diversity within their organizations.

### 1.2. Equal Employment Opportunity

Similar to AA, equal employment opportunity (EEO) policies comprise initiatives, policies or strategies which aim to accomplish better representation of excluded minorities in employment [30]. Moreover, both AA and EEO initiatives are based on moral and legal arguments, while diversity management (DM) differs by resting on a business case argument that a diverse workforce contributes to the organization’s performance and success [30,31]. More comprehensively, EEO is understood as anteceding AA, which in turn leads to DM [32].

Nonetheless, EEO and AA share similar outcomes when it comes to employees’ attitudes. Indeed, as proposed by Connell [10] in her study focused on the four dimensions of gender relationships—division of labor, relations of power, emotion and human relations, and culture and symbolism—whenever EEO had been used as a tool of organizational reconstruction, resentments were found on the male side and exasperation on the female side. Therefore, a predominant endorsement became evident toward “equal opportunity” but not to “affirmative action” at the organizational level [10].

Additionally, prior studies already suggested that although they do not reduce discrimination charges, the mere presence of EEO plans, offices, or committees did in fact increase employees’ rights awareness [13]. Hirsh and Kmec [13] argue, however, that such initiatives provide fewer effects in terms of filling positions than diversity management training programs. Such findings suggest that DM not only differs from AA and EEO in terms of their basis, but can also provoke more substantive results—either regarding business performance or rights awareness and charge fillings [13,30,31].

### 1.3. Diversity Management

Diversity management (DM) is understood as comprising those initiatives or actions that are not legally binding, where compliance is probable among companies which believe it is likely to improve corporate management [33]. The central idea of DM is based on the premise that promoting workforce diversity and inclusion leads to a nurturing and productive environment in which all are valued and contributive to the organizational success [12,34].

Indeed, DM encompasses practices considered vital to the inclusion of women. Such initiatives include, but are not limited to, recruitment, training and development, compensation, and management accountability policies [35,36]. Quite common also is the proposal of CEO commitment towards AA as a condition for appropriate DM and as a successful strategy for employing women and racial minorities [36,37].

Hence, there is agreement among authors that despite differences in terms of rhetoric and implementation, EEO and DM initiatives have historically proven to be inadequate to achieve their targets in the workplace [12,24,31,38,39]. It is argued that although AA cannot be related to the filing of discrimination charges, the mere presence of AA policies might raise employees’ rights awareness. That, in turn, increases the likelihood of disputes over discrimination (rather than reducing its occurrence) [13]. Nonetheless, prior investigations indicated that whenever effectively managed (via DM), workplace gender diversity not only leads to a positive organizational climate for women but also largely benefits the organization [36]. Scholars argue, however, that neither AA nor DM can be effective in achieving gender equity. At least, not without a relational and multilevel framework for managing diversity [12].

As stated earlier, the main objective of this study was to analyze the available literature on gender AA and management, particularly the antecedents of attitudes towards affirmative action for gender parity in organizations. To do so, we mapped the state of the art in contemporary management literature, aiming to uncover the main topics around gender AA in management discussed by scholars over the years, and what can be read in order to obtain the depth of understanding desired on this topic [40].

## 2. Materials and Methods

Seeking methodological and academic rigor, this SLR followed Tranfield et al. [41] by applying medical science methods to the management field in order to ensure it is both practitioner- and context-sensitive. The paper was developed in three stages, comprising the planning phase (Stage I), the protocol and execution phase (Stage II), and the analysis and report phase (Stage III). The first stage focused on delimiting the topic, the objectives, and this SLR research question: What is available in the literature about antecedents of attitudes towards affirmative action policies for gender parity in management? The second stage focused on developing the review protocol as well as operationalization of the review (database search selection and analysis). Lastly, the third stage included the thematic analysis, discussion, and report. 

Given the credibility ascribed to journals with the highest impact in the management field and containing validated knowledge [42,43], this SLR also followed Ribau et al. [44] and was based on a compilation of all published journal articles available in the SCOPUS database, excluding books, book chapters, or conference papers. As an international database, SCOPUS comprises a comprehensive, curated abstract and citation database of peer-reviewed publications with high impact, relevant and trusted research content from all over the world. This content is scrutinized with analytical tools that rank those scholar publications in percentiles and quartiles according to the different research fields. Moreover, it is extensively used worldwide by more than 300 academic, government and corporate institutions. Therefore, this database has been selected because it covers the most relevant and pertinent publications available on the topic under scrutiny in this study. Aiming to map the state of the art in contemporary management literature, only journals that published studies fitting the selection criteria were included. 

A review protocol was developed in order to assure the scientific rigor of the SLR, comprising three main steps: search criteria, assessment, and dataset structure. The first involved both the database selection and the search criteria guiding this paper. Therefore, a search was carried out for articles in the SCOPUS database including the words “affirmative action” and “manage*” (including variations such as managerial, manager, etc.) in the title, abstract, and/or keywords (step 1.1). The preliminary search resulted in a total of 449 articles; therefore, the search was further refined to articles published in journals on business, management and accounting (step 1.2), finally narrowing the results to studies including the keyword “gender” as a variable, antecedent, control and/or outcome (step 1.3). This final search reduced the dataset to 76 articles matching the aforementioned criteria. 

Afterwards, with the aim of identifying relevant literature on AA and management, a new screening process took place. Here, the articles’ keywords and abstracts were assessed based on: (A) the presence of “affirmative action” and “management” in the author’s keywords; and (B) abstract relevance or adherence to the topic (step 2.1). This assessment allowed a reasonable prioritization of 10% of the articles to be analyzed first (step 2.2), comprising those articles that matched both criteria (A + B). Subsequently, we read and assessed those studies scoring only in the presence of “affirmative action” and “management” in the author’s keywords (A) (step 2.3), followed by studies scoring only in abstract relevance assessment (B) (step 2.4) and finally the remaining articles with low matching criteria (scoring in neither A nor B). No exclusion criteria were adopted, although those 37 remaining articles (low matching) were only skimmed in order to identify pertinent information. The third step involved the dataset structure and analysis. Table 1 below summarizes the review protocol performed (a complete version of the protocol is available in Appendix A).

Finally, an interpretative synthesis was carried out following Ribau et al.’s [44] procedure. This consisted of an inductive derivation of the constructs, based mainly on this author’s understanding of the articles’ focus, core ideas and arguments. Thereupon, the data collected were organized in the previously designed dataset structure (step 3), consisting of a set of six tables: (1) Reading sheet; (2) Summary; (3) Papers by year; (4) Top publications; (5) Methodology and method; and (6) Summarized table of contents. The summary table developed during Stage II had been revised after steps 2.2, 2.3 and 2.4 in order to include the variables (antecedents and outcomes) most cited by the authors. After each revision, all articles previously entered were revisited and reviewed in order to consider the new variables.

As stated above, the aim of this study was to analyze the extant literature on gender AA and management, with a focus on understanding how scholars have approached the subject, while discussing attitudes towards affirmative action for gender parity in organizations. Therefore, we mapped the state of the art in contemporary management literature, uncovering the main topics around gender AA in management approached by scholars over the years. We focused particularly on the antecedents and outcomes discussed by the authors when investigating the relationship between gender AA and management.

## 3. Results and Discussions

The number of papers published per year remained relatively constant over the years, ranging between two and four publications. However, small increases in 1999 and 2013 were noted, with a peak in the number of publications being reached in 2014 (Table 2). The growth detected can be related to a reaction to non-governmental and international initiatives to strengthen and accelerate measures to achieve gender equality. In 1999, for instance, the UN promulgated the Gender Equality A/I “Special measures for the Achievement of Gender Equality” (ST/AI/1999/9) [45]. In 2013, the OECD Council published a Recommendation on Gender Equality in Education, Employment and Entrepreneurship [46], which might help explain the increase in the number of publications that led to a peak in 2014 (10.5%).

Studies in North America (51.3%) were predominant, followed by Australia (17.1%) and Africa (10.5%); countries sharing a history of ethnic issues, which led to an increased call for actions aiming to solve historical and discriminatory issues. Possibly as a response to the UN’s Gender Equality A/I [45], studies focused on North America tended to explore the potentially adverse reactions to the coercive nature of AA (as a governmental mandate), as well as the effectiveness of such diversity programs [28,37,47,48,49].

Australia, on the other hand, introduced the Australian Sex Discrimination Act in 1984, aiming for equal treatment and opportunity for men and women across the country. Besides protecting people from discrimination, the act aimed to make it “unlawful for people to be discriminated against in many areas of public life, including employment, education, buying goods, accommodation and housing, commonwealth laws and programs, and playing sport” [50]. In 1986, the AA (Equal Opportunity for Women) included private-sector corporations and universities to comply with the 1984 act [29,51,52]. In 1999, the act was further replaced by the national Equal Opportunity for Women in the Workplace (EOWW) Act [53], which required organizations with 100 or more employees to launch workplace plans with the aim of removing barriers to women’s access and progress in the company, making EOWW Agency the statutory authority to whom organizations should submit annual reports demonstrating compliance with the legislation [12,53,54,55]. Unsurprisingly, the number of studies published that same year (1999) increased. 

Nonetheless, 1999 marked the beginning of a trend in studies putting forward the argument that greater gender homogeneity within workgroups would lead to stronger group cohesion and member commitment while reducing interpersonal conflicts and turnover rates—in short, better performance through heterogeneity [56,57]. Gender AA regained attention as a topic of study after 2015 following the UN 2030 Agenda for Sustainable Development and the 17 goals which includes gender equity, further explaining the new growth in papers published. The concentration of studies, however, did not follow the previous trend, and attention was spread in a more balanced way among scholars in different regions of the globe.

In terms of the most popular sources, findings revealed a concentration of studies published in 13 journals. Over the past two decades (1999–2019), the leader in publications has been the Review of Public Personnel Administration (RPPA), which is focused on the effects of HR procedures, indicating a tendency to approach affirmative action investigation in a public sphere that expanded to organizational management. The other leading journals are Women in Management Review (WMR), the Journal of Business Ethics (JBE), Public Personnel Management (PPM), and the American Review of Public Administration (ARPA). A complete list of the top 13 publications on the topic is available in Appendix B.

Research on gender AA has been balanced between empirical and conceptual approaches. Over 50% of articles contained quantitative analysis and followed a positivist paradigm, mainly focused on causality and law-like generalizations [58]. Studies also come under the interpretivist paradigm (40.8%) with qualitative approaches, focused contexts, subjective meanings, and motivating actions. Worth noting is the lack of a mixed-method approach in studies (7.9%) in a pragmatic paradigm combining both observable phenomena and subjective meanings, which could enrich academic knowledge of the subject in a more comprehensive research strategy [59]. A potential gap in the literature is present here, providing future avenues of study for scholars and practitioners interested in investigating the complex social phenomena involved in plans involving affirmative action and attitudes towards it. Moreover, in an apparent attempt to enable in-depth investigation of affirmative action as a real-life contemporary phenomenon, case studies are quite frequent (39.5%) [59]. 

It is noteworthy that most studies took either general or gendered types of AA as their main topic of investigation (a detailed table with types of AA studies and their methods is available in Appendix C). A predominance of gender issues as the main topic was expected due to step 1.3 in the review protocol, which narrowed the search to studies that included gender as a variable, antecedent, control or outcome. However, the mere presence of ethnic main topics [26,48,60,61,62,63,64,65] and general AA studies—despite the filter performed to limit it to results within “gender” as a variable—indicated that gender permeates most investigations related to AA. Furthermore, gender appears to play a leading role even when it is not the primary objective of the research. Another potential gap to be explored by future studies arises from these findings, i.e., investigations considering gender as a moderator but also differences in attitudes towards AA when gender parity policies are the only ones in place, compared to general AA covering all minorities and diversity targets.

Finally, regarding the main topics covered by affirmative action and management research over the past 33 years, Table 3 presents a summary of all the constructs discussed, as well as their role as antecedents or outcomes.

There is a tendency among scholars to discuss an average of three constructs per article, with just a few discussing more than eight topics at the same time [29,31,63,66,67]. Another commonality among authors is the understanding of prior AA experiences [40], general perceptions of AA [25], and DM experience [21] as the most common antecedents. Similarly, scholars reached some agreement on affirmative action attitude [44]; prejudice, discrimination, tokenism, and stigmatization [24]; and performance [16] as the top outcomes of AA in management. Based on the interpretivist perspective we followed and given the congruence among scholars when investigating AA to discuss either antecedents or outcomes of such initiatives among employees—revealed in the present study—we opted to further scrutinize such topics separately, based on the articles’ focus, core ideas, and arguments. It is noteworthy that despite most frequently used by authors, the theoretical constructs “antecedents” and “outcomes” are not present in all studies. Nonetheless, we opted to adopt the terms in our analysis, in an inductive perspective. Therefore, the following sections address the most common topics discussed by scholars while investigating gender affirmative action in management.

### 3.1. Antecedents

#### 3.1.1. Affirmative Action Prior Experiences

Despite diverging on the conceptual definition of AA, EEO and DM (and the relationships among them), there is a noteworthy commonality among authors supporting prior experiences with one or all the aforementioned initiatives [22,23,66,68] as anteceding general perceptions [33,67,69,70,71] and attitudes towards them. More importantly, by considering the different initiatives as intertwined and connected to each other, scholars also pointed out the misunderstanding regarding the boundaries between the three as well as a spillover on the purpose or effect of each of them [23,28,29,31,33,34,47,68]. More recently, authors have agreed that it is precisely such blurred ideas on AA that may lead to misjudgment and rejection among individuals, particularly those that might feel threatened by such initiatives [72,73].

#### 3.1.2. General Perceptions of AA

Several authors also contend that public and private organizations differ in the drivers or motivations to initiate AA [22,31,56,64,65,74]. While the former responds to legal compliance [25,70], the latter can be motivated by the potential benefits of heterogeneity in management [12,50,54,61,75] or even understand this “as part of a design to achieve political goals” [33]. Still, prior experiences with AA influence employees and managers’ general perceptions and attitudes towards such initiatives [62,64,66,69]. 

There is agreement among authors that in order to manage diversity in the workplace efficiently, a thorough understanding of the influence of policies and procedures on the attitudes, perceptions and behaviors of employees (either targeted or affected by such policies) is required [22,23,25,66,69,74]. Leslie et al. [69], for instance, argue that perceptions of low self-competence and perceived stereotyping by others can negatively affect AA targets and self-evaluated performance. Foley and Williamson [22] further state that the success of gender parity initiatives is directly influenced by implicit bias both in training and hiring in relationship to AA. In a more positive approach, Choi and Rainey [23] argue that a combination of DM and organizational procedures perceived as fair leads to higher employee job satisfaction. Results also indicate women as having higher job satisfaction than their male counterparts whenever they perceive their organization as managing diversity appropriately and maintaining high organizational fairness [23]. 

Authors also agree on the positive influence of institutional and social forces on general perceptions of AA in the workplace [31,66]. However, particularly in terms of hiring processes and organizational issues stemming from AA (instead of the legislation itself), social interaction has a negative influence on attitudes towards AA [66].

#### 3.1.3. Merit

Divergent perceptions of gender AA in terms of merit are presented as an antecedent of attitude towards AA [31,35,55,66,71]. As mentioned before, Foley and Williamson [22] stated that implicit bias in AA could influence gender parity initiatives, which in turn would explain gender AA’s ineffectiveness in creating the cultural tipping point required to advance gender equality. However, such a change would not be feasible without a perilous reassessment of “merit” [22]. Moreover, although not directly questioning merit, the authors showed that individuals’ sensitivity to equity (or the perceived unfairness of gender AA programs) negatively influences the perception of recruitment based on AA policies [71]. Merit is also indicated as influencing managers’ attitudes towards identity-conscious (instead of identity-blind) activities in EEO and AA programs [35]. It is noteworthy, however, that despite being more frequently discussed as an antecedent, concerns over merit permeate every instance of affirmative action initiatives—either as violation or correction action [22,74,76]. 

**Figure 1:**
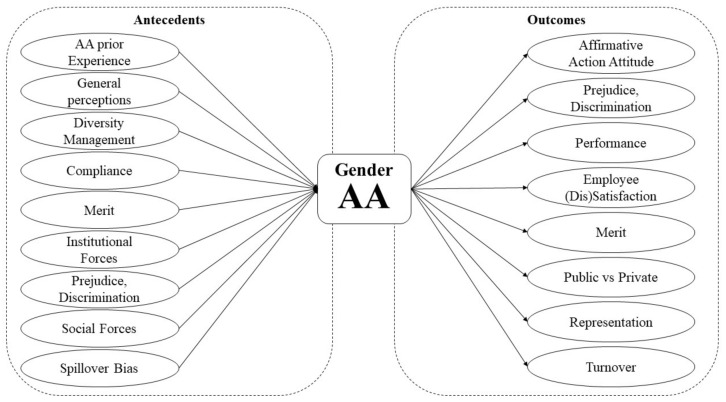
Path model of antecedents and outcomes of affirmative action in management.

Finally, gender appeared as either a control or moderator in some studies [23,24,63,66,77,78,79], further determining expectations and behaviors once gender AA is in place. As stated above, Choi and Rainey [23] pointed out that female employees tend to have higher job satisfaction when companies manage diversity effectively, combined with fair procedures. Oosthuizen et al. [63] also indicated the tendency among males to feel discriminated against by female competitors whenever AA is implemented. Such findings reinforce the idea that affirmative action—particularly gender AA—can be perceived differently by individuals of different genders [68]. Figure 1 illustrates the full path model developed in this paper, comprising the topics discussed by the authors, and mapping the relationship between antecedents and outcomes of AA in management.

### 3.2. Outcomes

#### 3.2.1. Attitudes towards AA

Following a more consistent trend, outcomes of gender AA in management are approached by scholars in a balanced and relatively aligned way. As expected, attitudes towards gender affirmative action as a combination of prejudice, discrimination, tokenism, and stigmatization [67,80] are the top topics presented. Scholars argue that divergent attitudes arise among individuals when they are the targeted (or excluded) party. Leslie et al. [69] further argue that stigmatization is not only recurrent in gender AA, but it can also ruin any efforts towards diversity management. Additionally, AA targets can be negatively affected by such initiatives even when they are not chronically stereotyped [69]. Hite [67] indicates that while AA targets (black respondents) tend to perceive discrimination/racism in their workplaces and are consequently supportive of affirmative action, non-targeted individuals (white participants) tend to have an illusion of equity, not perceiving racism in the workplace and failing to see the need for AA.

Alternatively, Oosthuizen et al. [63] proposed that not only do those employees affected by AA—but not targeted by it—perceive such initiatives as “reverse racism” but additionally, particularly when gender AA takes place, both black and white male employees report feeling discriminated against by female competitors. Indeed, the perception of being stereotyped due to AA has been reported by several employees across race and gender groups [63]. Additionally, scholars have reached some agreement that while employees understand the purposes of AA, men and women present negative attitudes when believing that practices discriminate against their own gender [48,63,68,69,81]. 

Investigating spillover bias both in diversity management judgement and decision-making, the experiments of Daniels et al. [68] have shown that individuals’ perceptions of diversity—and prejudice—can be inaccurate and, in turn, could hinder support for gender AA in corporations. Undeniably, attitudes towards AA can be observed in a myriad of ways, but special attention has been paid to variations in terms of unhappiness with how diversity issues are communicated and managed in the organization [22,64]. At the managerial level, for instance, where decisions on employees’ evaluation are challenging—they may vary within process and outcome accountability systems—political orientation can influence managers’ attitudes towards AA [82]. Indeed, political ideologies not only determine the type of preferred accountability systems (i.e., conservatives favor “outcome accountability” vs. “process accountability” preferred by liberals) but also in controversial initiatives such as AA. The split, however, is less distinct in affirmative action (demographic equality being the primary value) than in other controversial initiatives, such as gender AA [82]. 

Finally, when it comes to policies and strategies towards gender parity in corporate boards—and putting aside the merit of either hard or soft quotas—not only do such initiatives lead to stigmatization of those individuals targeted, but also the attitude towards such AAs can be equally (and diversely) influenced by such experiences [80]. Casey et al. [80] uphold that despite the presence of successful cases in advancing numerical gender parity in boards of governance—as in the Norwegian experience—such advancements can be greatly jeopardized if the stigma of women being appointed to such positions only to fulfil a legislative obligation persists.

#### 3.2.2. Performance

In terms of performance, gender and ethnic diversity have been under consistent scrutiny as significantly related to employee performance [28,31,34,69,77]. Although designed to enable workplace opportunities and development for members of target groups (e.g., women, ethnic minorities), AA has the ironic effect of stigmatizing those same targets [69], and in turn, lowering their performance outcomes [24,28]. In fact, the lack of competence and warmth on the non-targeted side influences their perception of AA targets’ performance (usually negative). Then again, self-competence and perceived stereotyping affects AA targets’ perceived performance [69]. The opposite is also true, because whenever non-targeted groups feel discriminated and undervalued, such feelings affect both the individual’s performance and that of the organization overall [24,31,56,69,77,83].

Consensus is yet to be found among authors in connecting affirmative action, diversity management, and performance. Nonetheless, gender and ethnic diversity have been positively and significantly related to performance—particularly in higher education institutions—the latter being the variable contributing most to improved employee performance. [77]. Moreover, empirical studies with top management teams did not indicate consistently increased performance when greater gender diversity was in place, although findings indicated that when the presence of female executive managers is less than 10%, the impact on firm performance is negative [56]. Conversely, diversity management initiatives that deal with stereotyping are indicated as enhancing performance—rather than affirmative action policies [83]. 

Indeed, Daniels et al. [68] argue that finding a consistent association between diversity and team performance is still a challenge. Their central argument is that because spillover bias affects diversity judgement, distorting answers and influencing attitudes, research on performance that considers how perceived diversity influences teams’ outcomes might allow “more robust scientific inferences about the outcome of diversity” [68] (p. 102).

#### 3.2.3. Employee Satisfaction

Authors have also connected employee satisfaction (after AA) to performance [23,29,30,84,85]. Noteworthy among the arguments is the reinforcing idea that a heterogeneous workgroup could create cultural synergy—among satisfied employees—that in turn would lead to increased performance [34]. Not only could the combination of diversity management and perceived fair organizational procedures increase employee job satisfaction, but women also tend to show higher job satisfaction when perceiving that the organization is maintaining fairness and managing diversity properly [23]. 

Authors argue that employees are more inclined to be satisfied when organizations provide not only a stable and harmonious workplace, but mainly when clear reward policies are provided, including AA [61]. Furthermore, findings indicate that employees in hotels with high and medium levels of ethnic diversity reported significantly higher levels of job satisfaction, as well as greater organizational commitment [75].

Nevertheless, despite the spread of initiatives aiming to increase women’s and minorities’ presence at firms’ top managerial levels, effective policies to achieve this without jeopardizing employees’ satisfaction are still causing heated debate [84]. Indeed, there is evidence that despite understanding the purposes of AA, all employees—irrespective of gender, age, language, tenure, and race—are unhappy with how diversity issues are communicated and managed in the organization [62]. Here, one must take into consideration that not only can gender AA itself compromise employee satisfaction, but also how diversity issues are communicated and managed in the organization [64]. There is still no understanding of which tools or strategies could efficiently improve employee satisfaction after gender AA.

#### 3.2.4. Merit

Finally, the relevance of meritocracy should be highlighted. Despite being underdiscussed by authors (only six occurrences), this occupies a bivalent position as both an antecedent and outcome of AA. Indeed, gender AA is commonly understood as either an unacceptable violation of merit [22,70,74,76,81,86], or as initiatives aimed at correcting the biased application of merit [22]. Nevertheless, even when gender AA is accepted as having the potential to address implicit biases, it still falls short in translating recognition of biased workplaces—and the need for diversity—into support for such initiatives, also failing to create the cultural “tipping point” to advance gender equality [22].

Particularly at the managerial level, gender AA is seen as either necessary measures to correct biased assessments of merit, or actively violating the core principles of merit—fairness, neutrality, and non-discrimination. In that case, when merit-correction AA is in place, managers tend to uphold an argument of the primacy of merit [22]. Moreover, findings indicate that when employees perceive gender AA preferential treatment (merit violation), negative job attitudes follow, particularly among those not favored [81]. Similarly, AA programs aimed at increasing cultural diversity were not only commonly seen by managers as having less priority, but also as being unfair, because they may favor some groups over the merit of others [76]. 

Nonetheless, most authors agree that what appears to be the most demanding action nowadays is a critical reassessment of merit [22,70,74,76,86]. That agreement indicates that despite the controversy surrounding gender affirmative action policies and their value in the public or private spheres, some convention has been reached among scholars.

## 4. Conclusions

Contemporary scholars agree that affirmative action initiatives in management, such as the use of gender quotas, can be highly controversial among employees, affecting them differently. Some are strongly supportive, recognizing the value of gender AA as a means to increase women’s descriptive representation and to correct biased selection and promotion programs; others are not supportive at all, arguing that such initiatives represent an unacceptable violation of merit. This systematic literature review explored the existing literature on management and gender affirmative action initiatives in organizations (public or private), in order to analyze what is available on the topic, particularly the way scholars discussed the antecedents of attitudes towards affirmative action for gender parity in organizations, as well as the most common outcomes of such initiatives among employees.

This systematic literature review covered 76 articles published over the past 33 years, providing not only the most recurrent topics on gender AA in management discussed by experts, but also mapping the most frequent antecedents and outcomes scrutinized by scholars. Notably, and following global initiatives, the topic gained increased attention in the years following UN goals and OECD recommendations, a growth not sustained over the following years. Studies were also mainly developed in regions and nations with a history of ethnic discrimination, as well as legal initiatives employed to compensate for societal barriers that prevented women and minorities from having equal access to employment opportunities and representation. The low number of publications per year demonstrates the lack of a persistent trend in investigating the issue, leaving a gap to be filled by future studies investigating gender AA policies and their effects on employee satisfaction and performance.

The research question persists: What is available in the literature about antecedents of attitudes towards affirmative action policies for gender parity in management? Our findings indicate that despite differing in motivations, public and private organizations share determinants of attitudes towards gender AA. Moreover, prior experiences and general perceptions of AA are the most common antecedents, while attitudes combining the “prejudice combo”—discrimination, tokenism, and stigmatization—as well as performance and employee (dis)satisfaction, are the typical outcomes of affirmative action in management. Gender also consistently appeared as either a control or moderator of such antecedents.

Therefore, some pertinent gaps in the literature were also presented, namely the lack of research considering gender as a moderator but also differences in attitudes towards AA when gender parity initiatives are the only ones in place, compared to general AAs covering all diversity targets. Notwithstanding, this paper brought the bivalent position of meritocracy—or the idea of merit violation—to light, as both an antecedent and outcome of AA. A critical reassessment of merit has been consistently demanded by scholars, indicating the latent need for empirical studies investigating not only the concept of merit but also its effect on employees’ and managers’ attitudes and policymaking.

Finally, it can be claimed that at least in terms of antecedents and outcomes of gender AA in management, there is a notable permeability or lack of strict boundaries between the causes and effects of such initiatives. This may indicate that a greater number and variety of studies are required, opening avenues of study for future scholars and practitioners interested in investigating the complex social phenomena involved in gender affirmative action plans and attitudes towards them.

### 4.1. Limitations of the Research

This SLR aimed at analyzing gender affirmative action in management and how scholars have approached the subject over time. Acknowledging that the topic remains a controversial subject among scholars, practitioners, and employees, this study refrained from scrutinizing the effectiveness of gender parity initiatives or AA policies, because it would have required a time series analysis. Furthermore, the analysis was held on a compilation of all published journal articles available on the SCOPUS database, which is less “elitist” than the Web of Science (WoS) scholar database. As such, a replication of this SLR procedure on the WoS database might be of added value. Finally, it is noteworthy that this paper did not aim at theory development regarding gender parity initiatives or AA policies. Instead, it proposed an analysis of the historical development of ideas and potential problem identification.

### 4.2. Future Studies

As mentioned above, the findings indicate that some gaps in the literature still need to be filled by future studies on gender AA policies and their effects on employee satisfaction and performance. For instance, the low number of publications per year demonstrates the lack of a persistent trend in investigating this issue. Moreover, longitudinal studies are in high demand because they could scrutinize changes over time, which could be of valuable contribution to the academia. Mixed-method studies, combining both observable phenomena and subjective meanings, could also enrich academic knowledge of the subject, in a more comprehensive research strategy to investigate the complex social phenomena involved in affirmative action plans and attitudes towards it. Finally, there is still little understanding on which tools or strategies could efficiently improve employee satisfaction when gender AA is in place. Future studies might benefit from the scrutiny of such relationships, as well as the role of meritocracy in both selection and promotion policies, that include or are developed under the diversity and inclusion management umbrella.

## Figures and Tables

**Table 1:** Systematic literature review Protocol.

Step	Description	Rationale	Total
1	Search criteria	Scopus	
	1.1 Preliminary search	keywords: (TITLE-ABS-KEY (“affirmative action”) AND TITLE-ABS-KEY (manage))	449
	1.2 Refined search	keywords: (TITLE-ABS-KEY (“affirmative action”) AND TITLE-ABS-KEY (manage)) AND (LIMIT-TO (DOCTYPE, “ar”)) AND (LIMIT-TO (SUBJAREA, “BUSI”)) AND (LIMIT-TO (SRCTYPE, “j”))	141
	1.3 Narrowed Refined search	keywords: ((TITLE-ABS-KEY (“affirmative action”) AND TITLE-ABS-KEY (manage))) AND (gender) AND (LIMIT-TO (SRCTYPE, “j”)) AND (LIMIT-TO (DOCTYPE, “ar”)) AND (LIMIT-TO (SUBJAREA, “BUSI”))	76
2	Assessment	Article screening	
	2.1 Article screening		76
	2.2 Read and assess first 10% papers	Studies that scored both in (A) and (B) on step 2.1	7
	2.3 Read and assess articles with keyword matches	Studies that scored only (A)	7
	2.4 Read and assess articles with abstract match	Studies that scored only (B)	37
	2.5 Skim articles with low match	Studies that neither scored (A) and (B)	25
3	Dataset Structure	Data compilation and analysis	

**Table 2:** Papers published by year.

			Region							
	Total		North America	South America	Europe	Asia	Australia	Africa	Cross-National: 2 Countries	Cross-National: +2 Countries
	# Studies	% of Studies	#	#	#	#	#	#	#	#
2019	4	5.3%	1				1	2		
2018	1	1.3%	1							
2017	2	2.6%	1			1				
2016	4	5.3%					2		1	1
2015	2	2.6%			2					
2014	8	10.5%	4			1	1	2		
2013	6	7.9%	4			1			1	
2012	3	3.9%	1		1	1				
2011	4	5.3%		2	1		1			
2010	4	5.3%	2			1	1			
2009	2	2.6%	1				1			
2008	2	2.6%	2							
2007	3	3.9%	3							
2006	4	5.3%	2		1			1		
2005	2	2.6%	1						1	
2004	3	3.9%	3							
2003	2	2.6%	2							
2002	0	0.0%								
2001	1	1.3%					1			
2000	3	3.9%					1	2		
1999	5	6.6%	4				1			
1998	1	1.3%					1			
1997	1	1.3%	1							
1996	1	1.3%						1		
1995	2	2.6%	2							
1994	2	2.6%	1				1			
1993	1	1.3%	1							
1992	0	0.0%								
1991	1	1.3%	1							
1990	1	1.3%	1							
1989	0	0.0%								
1988	0	0.0%								
1987	0	0.0%								
1986	1	1.3%					1			
	76	100.0%	39	2	5	5	13	8	3	1

**Table 3:** Topics covered.

Antecedents		Outcomes	
Affirmative Action prior Experience	40	Affirmative Action Attitude	24
General perceptions of AA	25	Prejudice, Discrimination, Tokenism and Stigmatization	24
Diversity Management	21	Performance	16
Compliance	13	Employee (Dis)Satisfaction	13
Merit (violation and/or correction)	8	Merit (violation and/or correction)	*6*
Institutional Forces	7	Public vs. Private	5
Prejudice, Discrimination	4	Representation (minorities presence)	5
Social Forces	4	Turnover	4
Spillover Bias	1		

## Data Availability

No new data were created or analyzed in this study. Data sharing is not applicable to this article.

## Appendix A

**Table A1:** Literature review: full protocol.

Step	Description	Rationale	Total
**1**	Database Search definition	The review will use data from Scopus once it comprises publications with higher impact factors	
	1.1 Preliminary search—keywords: (TITLE-ABS-KEY (“affirmative action”) AND TITLE-ABS-KEY (manage))	In order to identify relevant general literature on AA and management (including variations such as managerial, manager, etc.)	449
	1.2 Refined search—keywords: (TITLE-ABS-KEY (“affirmative action”) AND TITLE-ABS-KEY (manage)) AND (LIMIT-TO (DOCTYPE, “ar”)) AND (LIMIT-TO (SUBJAREA, “BUSI”)) AND (LIMIT-TO (SRCTYPE, “j”))	Refine search to articles published in journals on Business, Management, and Accounting areas	141
	1.3 Narrowed Refined search—keywords: ((TITLE-ABS-KEY (“affirmative action”) AND TITLE-ABS-KEY (manage))) AND (gender) AND (LIMIT-TO (SRCTYPE, “j”)) AND (LIMIT-TO (DOCTYPE, “ar”)) AND (LIMIT-TO (SUBJAREA, “BUSI”))	Narrow search to studies that include gender as both variable, antecedent, control and/or outcome	76
**2**	Assessment	Articles were sorted from most recent to least recent and numbered accordingly.	
	2.1 Article screening	Assess articles based on the presence of “affirmative action” and “management” in the author’s keywords (A) and abstract relevance assessment (B)	76
	2.2 Read and assess first 10% of papers	Studies that scored both in the presence of “affirmative action” and “management” in the author’s keywords (A) and abstract relevance assessment (B) on step 2.1 were selected to be the first entered/analyzed	7
	2.3 Read and assess articles with keyword matches	Studies that scored only in the presence of “affirmative action” and “management” in the author’s keywords (A)	7
	2.4 Read and assess articles with abstract matches	Studies that scored only in abstract relevance assessment (B) were read and accessed	37
	2.5 Skim articles with low match	Studies that scored neither in the presence of “affirmative action” and “management” in the author’s keywords (A) or abstract relevance assessment (B) were skimmed in order to identify potentially relevant information	25
**3**	Dataset Structure	All data compiled from the articles were analyzed and classified by year, source, methodology, topic, antecedents/outcomes.	

## Appendix B

**Table A2:** Top 13 publications ^1^.

Number of Articles Published per Source (per Year) *														
		RPPA	WMR	JBE	PPM	ARPA	SAJHRM	OBHDP	MS	EDI	PPM	IR	IJHRM	GWO
No. of Studies **		8	5	3	3	3	2	2	2	2	2	2	2	2
	TT/Year ***	10.5%	17.1%	21.1%	25.0%	28.9%	31.6%	34.2%	36.8%	39.5%	42.1%	44.7%	47.4%	50.0%
2019	4	1					2							
2018	1			1										
2017	2							1						
2016	4													
2015	2								1	1				
2014	8	1			1						1			
2013	6				1			1	1	1				
2012	3	1										1	1	
2011	4													1
2010	4	1												1
2009	2											1		
2008	2										1			
2007	3					1								
2006	4	1	2											
2005	2													
2004	3	2												
2003	2													
2002	0													
2001	1													
2000	3		2		1									
1999	5	1		1									1	

Notes: ***** only the top 13 journal outlets with higher numbers of publications over the past two decades (1999–2019) are presented. ** Total number of studies published per journal. *** Total number of articles published per year. ^1^ Source Acronyms: Review of Public Personnel Administration (RPPA); Women in Management Review (WMR); Journal of Business Ethics (JBE); Public Personnel Management (PPM); American Review of Public Administration (ARPA); SA Journal of Human Resource Management (SAJHRM); Organizational Behavior and Human Decision Processes (OBHDP); Management Science (MS); Equality, Diversity and Inclusion (EDI); Problems and Perspectives in Management (PPM); Industrial Relations (IR); International Journal of Human Resource Management (IJHRM); Gender, Work and Organization (GWO).

## Appendix C

**Table A3:** Main Methods and Types of Study.

		Methods						Type of AA					
		Qualitative		Quantitative		Mixed-Method		Ethnic/Racial		Gender		General Diversity	
No. of Studies		#	%	No. of Studies	% of Studies	No. of Studies	% of Studies	No. of Studies	% of Studies	No. of Studies	% of Studies	No. of Studies	% of Studies
2019	4	2	2.6%	2	2.6%			2	2.6%	1	1.3%	1	1.3%
2018	1	1	1.3%		0.0%							1	1.3%
2017	2			2	2.6%								
2016	4	4	5.3%									2	2.6%
2015	2	1	1.3%	1	1.3%					2	2.6%		
2014	8	2	2.6%	5	6.6%	1	1.3%	3	3.9%	1	1.3%	4	5.3%
2013	6	4	5.3%	2	2.6%					2	2.6%	4	5.3%
2012	3	1	1.3%	2	2.6%			1	1.3%			2	2.6%
2011	4	1	1.3%	3	3.9%			1	1.3%	2	2.6%	1	1.3%
2010	4	1	1.3%	3	3.9%					2	2.6%	2	2.6%
2009	2	1	1.3%	1	1.3%							2	2.6%
2008	2	1	1.3%	1	1.3%			1	1.3%			1	1.3%
2007	3	2	2.6%	1	1.3%							3	3.9%
2006	4	2	2.6%	1	1.3%	1	1.3%			3	3.9%	1	1.3%
2005	2			2	2.6%					2	2.6%		
2004	3	1	1.3%	1	1.3%	1	1.3%					3	3.9%
2003	2	1	1.3%			1	1.3%					2	2.6%
2002	0												
2001	1			1	1.3%					1	1.3%		
2000	3	1	1.3%	2	2.6%					2	2.6%	1	1.3%
1999	5	1	1.3%	4	5.3%			2	2.6%	1	1.3%	2	2.6%
1998	1	1	1.3%									1	1.3%
1997	1			1	1.3%					1	1.3%		
1996	1	1	1.3%							1	1.3%		
1995	2					2	2.6%			1	1.3%	1	1.3%
1994	2			2	2.6%					2	2.6%		
1993	1			1	1.3%							1	1.3%
1992	0												
1991	1	1	1.3%									1	1.3%
1990	1	1	1.3%									1	1.3%
1989	0												
1988	0												
1987	0												
1986	1			1	1.3%					1	1.3%		
	76	31	40.8%	39	51.3%	6	7.9%	10	13.2%	25	32.9%	37	48.7%
